# Population pharmacokinetics of ivermectin for the treatment of scabies in Indigenous Australian children

**DOI:** 10.1371/journal.pntd.0008886

**Published:** 2020-12-07

**Authors:** Amanda Gwee, Stephen Duffull, Xiao Zhu, Steven Y. C. Tong, Noel Cranswick, Brett McWhinney, Jacobus Ungerer, Joshua Francis, Andrew C. Steer

**Affiliations:** 1 Department of General Medicine, The Royal Children’s Hospital Melbourne, Parkville, Victoria, Australia; 2 Department of Paediatrics, The University of Melbourne, Parkville, Victoria, Australia; 3 Infection and Immunity theme, Murdoch Children’s Research Institute, Parkville, Victoria, Australia; 4 School of Pharmacy, University of Otago, Dunedin, New Zealand; 5 Victorian Infectious Diseases Service, The Royal Melbourne Hospital, and Doherty Department University of Melbourne, at the Peter Doherty Institute for Infection and Immunity, Victoria, Australia; 6 Global and tropical health division, Menzies School of Health Research, Charles Darwin University, Darwin, Australia; 7 Department of Chemical Pathology, Pathology Queensland, Brisbane, Queensland, Australia; 8 Faculty of Biomedical Science, University of Queensland, Brisbane, Queensland, Australia; 9 Department of Paediatrics, Royal Darwin Hospital, Northern Territory, Australia; Hebrew University Hadassah Medical School, ISRAEL

## Abstract

Ivermectin is a broad-spectrum antiparasitic agent used for the treatment and control of neglected tropical diseases. In Australia, ivermectin is primarily used for scabies and is licensed in children aged ≥5 years weighing >15 kg. However, young children, aged <5 years, are particularly vulnerable to scabies and its secondary complications. Therefore, this study aimed to determine an appropriate ivermectin dose for children aged 2 to 4 years and weighing ≤15 kg. We conducted a prospective, pharmacokinetic study of ivermectin in Indigenous Australian children aged between 5 and 15 years and weighing >15 kg. Doses of 200 μg/kg rounded to the nearest whole or half 3 mg tablet were given to children with scabies and ivermectin concentrations determined at two time points after dosing. A population pharmacokinetic model was developed using non-linear mixed effects modelling. A separate covariate database of children aged 2 to 4 years and weighing <15 kg was used to generate 1000 virtual patients and simulate the dose required to achieve equivalent drug exposure in young children as those aged ≥5 years. Overall, 26 children who had 48 ivermectin concentrations determined were included, 11 (42%) were male, the median age was 10.9 years and median body weight 37.6 kg. The final model was a two-compartment model with first-order absorption and linear elimination. For simulated children aged 2 to 4 years, a dose of 3 mg in children weighing 10–15 kg produced similar drug exposures to those >5 years. The median simulated area under the concentration-time curve was 976 μg∙h/L. Using modelling, we have identified a dosing strategy for ivermectin in children aged 2 to 4 years and weighing less than 15 kg that can be prospectively evaluated for safety and efficacy.

## Author summary

Ivermectin is an important drug for the control and treatment of neglected tropical diseases. However, despite numerous studies showing that ivermectin is safe and well tolerated in young children, it is not currently recommended in young children <5 years and <15 kg. Therefore, there are no guidelines for the dose of ivermectin in young or small children. In this study, we firstly determined how much ivermectin is present in blood in children aged 5 years and older. We then used this information to model what happens to ivermectin in childrens’ bodies allowing us to calculate the dose required in children aged less than 5 years and weighing under 15 kg. This study provides a new dosing guideline that can now be tested in clinical studies of children <5 years and <15 kg.

## Introduction

Ivermectin is a broad-spectrum antiparasitic agent used for the treatment and control of onchocerciasis, lymphatic filariasis and soil-transmitted helminths including strongyloidiasis[1]. In Australia, ivermectin is used to treat scabies infection and is indicated as first-line therapy for crusted scabies and as second-line for typical scabies when topical permethrin has failed[2]. Scabies infestation causes considerable morbidity through itch, scratch and stigma, and predisposes to secondary infection with *Streptococcus pyogenes* and *Staphylococcus aureus*, which in turn can be complicated by more severe conditions including deep tissue infections, sepsis, post-streptococcal glomerulonephritis and rheumatic heart disease[3].

Ivermectin has emerged as a highly effective treatment for scabies when used in whole communities as part of public health efforts to control the disease[3]. There is a vast amount of experience in the use of ivermectin as part of mass drug administration for control of onchocerciasis and lymphatic filariasis. However, these public health strategies do not recommend the drug for young children. The pharmacodynamic target of ivermectin for scabies is poorly understood although higher drug exposure as measured by the area under the concentration-time curve has been shown to improve outcomes for other tropical infections[4].

Ivermectin is currently licensed in Australia for use in children aged five or more years and weighing greater than 15 kg. However, young children aged less than five years are at high risk of scabies infestation and its secondary complications[5]. Available data suggest that ivermectin is safe and well tolerated in young children, but further studies are needed to determine the appropriate dose in this group[6]. Therefore, in this study we conducted a prospective pharmacokinetic study to develop a population pharmacokinetic model of ivermectin in children in whom the drug is currently licensed and used the model to simulate an appropriate dose for children aged between two and four years and weighing less than 15 kg.

## Methods

### Ethics statement

The study was approved by the Human Research Ethics Committee of Northern Territory Department of Health and Menzies School of Health Research (HREC 2015–2519).

The Ivermectin Therapy in Children (ITCH) study was an open label, prospective, pharmacokinetic study of ivermectin in Indigenous Australian children aged between five and 15 years and weighing over 15 kg. Children were enrolled in a remote community in Arnhem Land in the Northern Territory of Australia with a population of over 2000 people between 1 April 2016 and 31 March 2018[7].

Children with either crusted scabies or scabies that failed to respond to topical therapy requiring ivermectin were included. Those with known liver impairment, allergy to ivermectin, treatment with warfarin or a neurological disease were excluded. Informed consent was obtained from the parent or guardian by a member of the study team prior to any study procedure. Ivermectin was given at doses of 200 μg/kg rounded to the nearest whole or half 3 mg tablets. Each dose was directly observed and administered with food.

Ivermectin concentrations were determined by blood sampling at two time points after dosing with five blood sampling-design groups, in order to determine plasma concentrations distributed over the concentration-time curve (Table 1). The sampling-design groups were optimised using POPT (optimal design software) based on the prior pharmacokinetic model[8]. Equal numbers of children were allocated to each of the five groups, although parents were able to select their preferred group. Samples were centrifuged immediately after blood draw and stored at 3°C on-site in a temperature-monitored refrigerator. Samples were then transferred on dry ice and stored at -80°C in Pathology Queensland, Australia.

**Table 1:** Timing of blood samples in the 5 groups.

Group	Sample time after first dose	
	Sample 1	Sample 2
1	2–6 hours	6–8 days
2	6–12 hours	8–10 days
3	3–24 hours	2–4 days
4	12–48 hours	10–12 days
5	2–5 days	12–14 days

The plasma ivermectin concentration was measured by reverse phase isocratic Ultra Performance Liquid Chromatography (UPLC) coupled with Fluorescence Detection (Waters Corporation, Milford, MA, USA). Inter-run imprecision across three levels of quality control was <8%. The lower limit of quantification was 2 μg/L and the upper limit was 200 μg/L.

Data including patient age, sex, body weight, dose received, medical history, clinical response to treatment, and treatment-related adverse effects (cutaneous, neurological, gastrointestinal) were recorded in a REDCAP® database by the treating clinician. Patients were reviewed two weeks after the first ivermectin dose by the treating clinician to assess clinical response and to administer a second ivermectin dose to complete the treatment course. Clinical response was categorised as unchanged, improved or worsened based on examination findings and patient or parent/guardian report.

Data were analysed using a non-linear mixed effects modelling approach in NONMEM version 7.3 (NONMEM®, ICON Development Solutions, Ellicott City, MD; with modelling workbench Pirana 2.9.7)[9]. The first order-conditional estimation with interaction (FOCEI) algorithm was used during model development, while, FOCEI followed by stochastic approximation expectation-maximisation (SAEM) algorithm was used for parameter estimation of the final model[10]. Model development involved identification of the structural model (one versus two compartment model) and models for between-subject variability and residual error. Covariates were tested that were deemed biologically relevant (weight, age, sex). For continuous covariates, a power model was considered (with and without fixing the exponent to standard allometric values). For the discrete covariate sex, females were taken to be the reference and the influence of males was estimated. It was anticipated that the number of children and number of samples would be small and therefore we also considered fixing the exponent for clearance and volume to a biological prior (i.e. to 0.75 for clearance and 1 for volume)[11]. As the aim of the current study was to extrapolate (by simulation) an appropriate dose for younger children different from the study population, preference was given to the choice of a biological prior. Model performance was evaluated using goodness-of-fit plots and the prediction-corrected visual predictive check (pcVPC) using PsN 4.8.1[12].

We used a separate covariate database of 288 indigenous and non-indigenous Australian children aged between two and four years and weighing less than 15 kg to generate 1000 virtual patients and simulate 1000 concentration-time profiles (one per patient) based on the developed population pharmacokinetic model. The area under the concentration-time curve (AUC) was calculated using trapezoidal integration over the concentration-time profile for each patient. Two different dosing scenarios were evaluated with: (i) a dose of 200 μg/kg, rounded to the nearest half or whole 3 mg tablet; and (ii) one 3 mg tablet, as suggested by a recent publication for children weighing less than 15 kg[13]. Simulations were performed in R version 3.5.3 to determine the dose required to achieve an equivalent exposure (AUC) in children aged between two and four years and weighing less than 15 kg to those aged five to 15 years and weighing at least 15 kg. Equivalent exposure was defined as a median ivermectin AUC in simulated children within the 80% - 125% range of the observed median AUC from children enrolled in the current study.

## Results

We enrolled 26 children; 11 (42%) were male, the median age was 10.9 years (range 5.5 to 14.9) and median body weight was 37.6 kg (18.5 to 74.5). Indications for ivermectin therapy were failure of topical therapy in 24 participants (92%), suspected allergy to permethrin in one (4%) and severe scabies with secondary bacterial infection in one (4%). The median dose given was 190 μg/kg (range 120 to 230) and there were no drug-related adverse effects. Twenty-two children attended their two-week follow-up appointment and at this time, 11 (50%) had complete resolution of their infection, eight (36%) partial resolution, and three (14%) had no improvement.

The 26 children had 48 ivermectin concentrations measured. The median number of ivermectin concentrations measured per patient was 2 (range 1 to 2). The final model was a two-compartment model with first-order absorption and linear elimination. Between-subject variability was included on clearance (*CL*), intercompartmental clearance (*Q*) and both volume parameters (volume of the central compartment (*V_c_*) and volume of the peripheral compartment (*V_p_*)) with an exponential residual error model. The best model incorporated weight as a covariate on both *CL* and *V_c_*. No other covariates were found to improve the fit. Two models were created, one with fixed exponents (at the biological prior) and one with estimated exponents. Although the model with the estimated exponents provided a statistically better fit for the data (with a lower objective function value), it provided the same inference in terms of dose prediction to the model with biological priors. The model with biological priors was therefore considered to be the final model and used as the basis for extrapolated dose-exposure simulations. For completeness, both models are provided in the supporting information with pcVPCs (S1 Text, S2 Text, S1 Table, S2 Table and S1 Fig) and the predicted exposure for different weight groups using both models compared (S2 Fig). Shrinkage of the individual estimates of *CL* (used to determine AUC) was low (S1 Table and S2 Table) indicating that individual predicted exposures calculated from individual values of dose and *CL* are anticipated to be unbiased.

**Fig 1:**
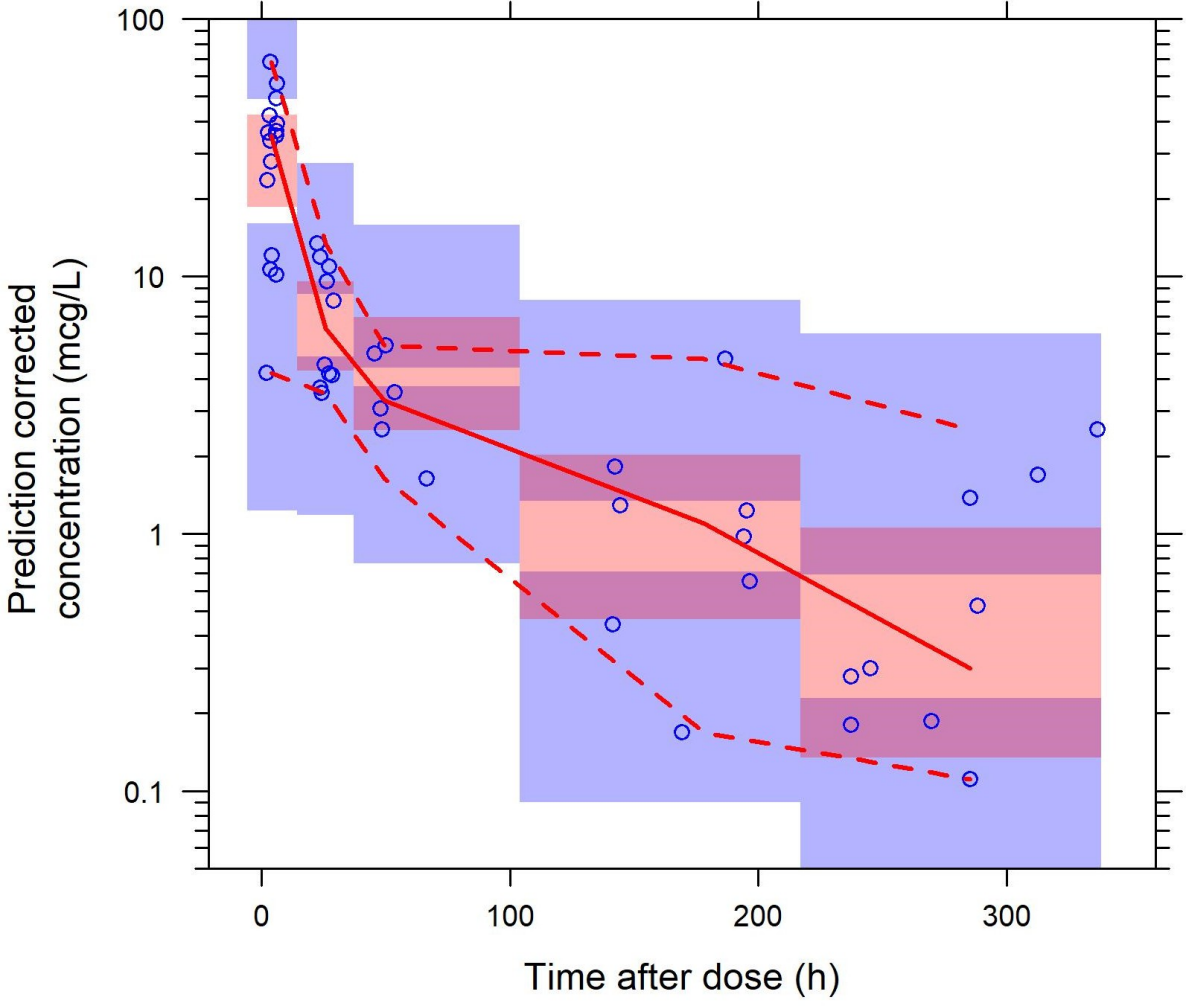
Prediction-corrected visual predictive check. The open circles are observed concentrations. The dashed lines are the 10^th^ and 90^th^ percentiles of the model predictions and the solid line is the 50^th^ percentile. The bands are the 95% CI of the percentiles of the model predictions.

From the final model, a typical individual of 37.6 kg had a clearance of 6.94 L/h (residual standard error (RSE) 18%), and clearance was found to increase with body weight with the exponent for clearance fixed to the biological prior 0.75. In the model, body weight was also incorporated as an important covariate on *V_c_* with the exponent fixed to its biological prior 1. The goodness-of-fit plots and prediction-corrected visual predictive check (pcVPC) showed good model performance (Fig 1 for pcVPC, and S1 Table for the model parameter estimates).

**Fig 2:**
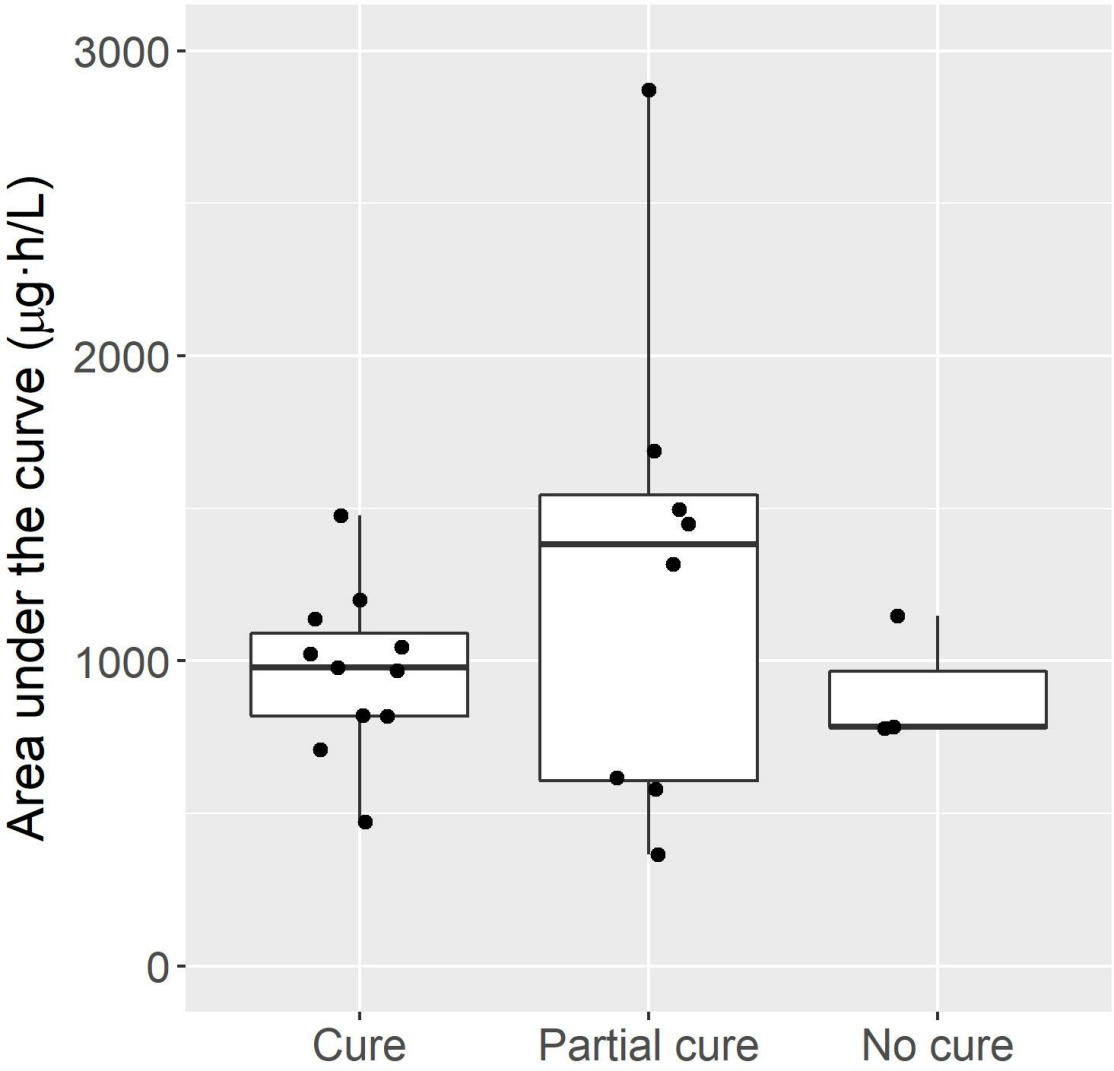
Drug exposures (area under the curve) vs clinical responses from children enrolled in the current study.

The median AUC was 1001 (IQR 727–1228) μg∙h/L using data from enrolled children. Drug exposure (AUC) was similar in children aged between 5 and 11 years and those aged over 11 years with median AUC values of 895 (IQR 728–1039) μg∙h/L and 1173 (IQR 740–1350) μg∙h/L, respectively (independent two-group t-test, p = 0.270). There was no difference in the AUC in those that achieved cure, partial cure, and no response to treatment (Fig 2, one-way ANOVA, p = 0.358).

For simulated children aged 2 to 4 years, doses of 200 μg/kg (rounded to the nearest half or whole 3 mg tablet, i.e. 1.5 mg for 10–11 kg, 3 mg for 12–15 kg) produced median AUC values of 917 (IQR 626–1312) μg∙h/L. However, for children weighing less than 11 kg, this dose led to lower drug exposures than those observed in the study group with a median AUC of 620 (IQR 469–849) μg∙h/L. A recently published dosing strategy of one 3 mg tablet for children weighing less than 15 kg was also evaluated[13]. This dosing regimen resulted in median AUC of 976 (IQR 671–1384) μg∙h/L. The median simulated AUC for children weighing 10–11 kg and 12–15 kg were 1240 (IQR 938–1699) and 953 (IQR 649–1357) μg∙h/L respectively. Therefore, a dose of 3 mg for children weighing 10 to 15 kg achieved median simulated drug exposures within the 80%-125% range of the observed median AUC for the older cohort of children enrolled in the study.

## Discussion

Ivermectin is an increasingly important drug for prevention and treatment of neglected tropical diseases and scabies[14–16]. Recently, the repurposing of ivermectin for the control of malaria has been identified as a public health priority due to its mosquitocidal activity[17]. For many of these diseases, in particular scabies, young children in resource poor settings are especially vulnerable to infection and associated secondary complications. Therefore, improving access to ivermectin for these children is important for public health. This study identified a dosing strategy (i.e. a dose of 3 mg) for children aged 2 to 4 years that achieves comparable drug exposure to children aged over 4 years. Our results are similar to a prospective pharmacokinetic study of children in Cote d’Ivoire that recommended a dose of 3 mg in children weighing 10 to 15 kg[13,18]. We propose that our simulated dose of 3 mg can now be prospectively evaluated to determine safety and efficacy in children aged 2 to 4 years and weighing 10 to 15 kg.

The target drug exposure (AUC) required to maximise the anti-parasitic effect of ivermectin against a range of target parasites has not been determined, including scabies and soil-transmitted helminths. However, a recent study of *Trichuris trichuria* showed an increase in the odds of cure with higher drug exposure as measured by the area under the concentration-time curve (AUC_0-∞_) [4]. In contrast, our study showed no difference in AUC in those who were cured of their scabies infestation compared to those who did not respond to treatment. However, our study was small and we used an imprecise measure of cure (clinical examination) as opposed to objective measurements of the scabies parasite[19]. Prior pharmacokinetic studies in adults have not determined the effect of drug exposure on treatment outcome for other parasites[20–22]. Nonetheless, community effectiveness of two doses of 200 μg/kg of ivermectin using mass drug administration for scabies control has been well described[23].

Our study results are similar to the only other pharmacokinetic study of ivermectin in young children. That study of children living in rural Cote d’Ivoire with *Trichuris trichuria* infection, showed that doses of 300 μg/kg were required in children aged 2 to 5 years[13]. Previous pharmacokinetic studies in both adults and children have found body weight to be the only significant covariate influencing ivermectin pharmacokinetics, as we found in our study[13,20]. An open-label pharmacokinetic study of 12 adult volunteers studied multiple covariates including age, sex, height, body weight, body surface area, percentage body fat, gall bladder volume, kidney volume, liver volume, haematocrit, plasma albumin, and MDR1 polymorphism (associated with lower P-glycoprotein pump expression in the intestinal tract) and found only body weight to be significant[20]. The only other study of ivermectin pharmacokinetics in children reported a clearance/kg of 0.35 L/h/kg in children, similar to our results (0.18 L/h/kg) with the difference likely explained by different study populations[13].

The primary safety concern of ivermectin treatment in young children is the risk of neurotoxicity. However, a review of nine studies where ivermectin was administered to children aged less than five years and weighing less than 15 kg (107 patients in total) found no serious drug-related adverse effects associated with ivermectin use with doses between 150 and 200 μg/kg[6,24–32]. Furthermore, in a cohort of 170 infants and children aged between one and 64 months treated with ivermectin at a mean dose of 223 μg/kg, no serious adverse effects were reported[33]. Ivermectin has a large therapeutic window. A study of adults that evaluated doses 10 times the recommended dose of 200 μg/kg reported no neurotoxicity[34]. Furthermore, two large trials of mass drug administration of oral ivermectin to 21,444 and 14,556 adults and children have shown that ivermectin is not associated with serious adverse events[15,35]. For children aged less than five years who typically cannot swallow tablets, the tablet can be administered dispersed or crushed[36]. However, if an ethanol-based solution is used, a previous study reported up to double the bioavailability of ivermectin (AUC ratio 1.08–2.29)[37].

The main limitation of this study was the small sample size and number of samples per patient. Also, there were limited data on the absorption phase and therefore we could not estimate the absorption rate constant. However, data were collected prospectively, and staff were trained to accurately document the timing of doses and blood samples. Also, our final model and simulated doses were similar to another prospective pharmacokinetic study in children.

Using population pharmacokinetic modelling, we identified a dosing strategy for ivermectin in children aged 2 to 4 years and weighing less than 15 kg—a dose of 3 mg in children weighing 10 to 15 kg. Young children in resource-poor settings are at especially high risk of infection with multiple parasites susceptible to ivermectin, including scabies, and an appropriate dosing strategy has the potential to confer substantial health benefits for this high-risk population.

## Supporting information

S1 Text Model with biological prior. (DOCX)

S2 Text Model with estimated exponents. (DOCX)

S1 Table Parameter estimates of the model with biological prior. (DOCX)

S2 Table Parameter estimates of the model with estimated exponents. (DOCX)

S1 Fig Prediction-corrected visual predictive check for the model with estimated exponents. The open circles are observed concentrations. The dashed lines are the 10^th^ and 90^th^ percentiles of the model predictions and the solid line is the 50^th^ percentile. The bands are the 95% CI of the percentiles of the model predictions.
(TIF)

S2 Fig Comparison of drug exposure (AUC) with current dose regimen (200 μg/kg) in the study population (children aged between 5 and 15 years and weighing >15 kg) using both the model with estimated exponents and the model with biological prior. (TIF)
