# Supplementary material for: Population pharmacokinetics of ivermectin for the treatment of scabies in Indigenous Australian children
Source: PLoS Negl Trop Dis. 2020 Dec 7;14(12):e0008886. doi: 10.1371/journal.pntd.0008886 (PMC7746298; doi:10.1371/journal.pntd.0008886)
Supplement: S2 Text — (DOCX) [file pntd.0008886.s002.docx]

Model with estimated exponents:

$$CL/F\left( L/h \right)=6.73\cdot\left( \frac{Weight}{37.55} \right)^{0.944}$$

$$V_{c}/F\left( L \right)=160\cdot\left( \frac{Weight}{37.55} \right)^{2.16}$$

$$Q/F\left( L/h \right)=2.95$$

$$V_{p}/F\left( L \right)=447$$
