# Supplementary material for: Population pharmacokinetics of ivermectin for the treatment of scabies in Indigenous Australian children
Source: PLoS Negl Trop Dis. 2020 Dec 7;14(12):e0008886. doi: 10.1371/journal.pntd.0008886 (PMC7746298; doi:10.1371/journal.pntd.0008886)
Supplement: S1 Table — (DOCX) [file pntd.0008886.s003.docx]

S1 Table. Parameter estimates of the model with biological prior

| **Parameters** | **Estimates (RSE%) [Shrinkage%]** |
| --- | --- |
| Typical value of clearance $(TVCL, L/h)$ | 6.94 (18%) |
| Typical value of central volume $(TVV_{c}, L)$ | 88 (53%) |
| Typical value of intercompartmental clearance ($TVQ, L/h)$ | 13.7 (14%) |
| Typical value of peripheral volume ($TVV_{p}, L$) | 344 (14%) |
| Absorption rate constant (${TVk}_{a}, 1/h$) | 0.5 fixed |
| Exponent for body weight on clearance ($\theta_{WT,CL}$) | 0.75 fixed |
| Exponent for body weight on central volume ($\theta_{WT,Vc}$) | 1 fixed |
| Exponent for body weight on clearance ($\theta_{WT,Q}$) | 0.75 fixed |
| Exponent for body weight on central volume ($\theta_{WT,Vp}$) | 1 fixed |
| Between subject variability (%CV)  CL  Vc  Q  Vp  Correlation (CL, Vc)  Correlation (CL, Q)  Correlation (CL, Vp)  Correlation (Vc, Q)  Correlation (Vc, Vp)  Correlation (Q, Vp) | 54% (25%) [10%]  237.1% (36%) [18%]  40.8% (44%) [22%]  38.9% (43%) [18%]  -30%  70%  86.3%  46.7%  21.5%  96.4% |
| Residual error  $\sigma_{additive}$ in log domain | 0.198 [41.8%] |

$\sigma_{additive}$ – standard deviation of the additive component of the residual error
