# Supplementary material for: Population pharmacokinetics of ivermectin for the treatment of scabies in Indigenous Australian children
Source: PLoS Negl Trop Dis. 2020 Dec 7;14(12):e0008886. doi: 10.1371/journal.pntd.0008886 (PMC7746298; doi:10.1371/journal.pntd.0008886)
Supplement: S2 Table — (DOCX) [file pntd.0008886.s004.docx]

S2 Table. Parameter estimates of the model with estimated exponents

| **Parameters** | **Estimates (RSE%) [Shrinkage%]** |
| --- | --- |
| Typical value of clearance $(TVCL, L/h)$ | 6.73 (23%) |
| Typical value of central volume $(TVV_{c}, L)$ | 160 (28%) |
| Typical value of intercompartmental clearance ($TVQ, L/h)$ | 2.95 (37%) |
| Typical value of peripheral volume ($TVV_{p}, L$) | 447 (10%) |
| Absorption rate constant (${TVk}_{a}, 1/h$) | 0.5 fixed |
| Exponent for body weight on clearance ($\theta_{WT,CL}$) | 0.944 (3%) |
| Exponent for body weight on central volume ($\theta_{WT,Vc}$) | 2.16 (4%) |
| Between subject variability (%CV)  CL  Vc  Q  Vp  Correlation (CL, Vc)  Correlation (CL, Q)  Correlation (CL, Vp)  Correlation (Vc, Q)  Correlation (Vc, Vp)  Correlation (Q, Vp) | 68.7% (26%) [7%]  86.9% (48%) [16%]  108.7% (37%) [15%]  29.8% (28%) [7%]  -4.2%  39.9%  -95.8%  -93.4%  -23.9%  -12.5% |
| Residual error  $\sigma_{additive}$ in log domain | 0.062 (56.1%) |

$\sigma_{additive}$ – standard deviation of the additive component of the residual error
