# Supplementary figures and images for: Population pharmacokinetics of ivermectin for the treatment of scabies in Indigenous Australian children
Source: PLoS Negl Trop Dis. 2020 Dec 7;14(12):e0008886. doi: 10.1371/journal.pntd.0008886 (PMC7746298; doi:10.1371/journal.pntd.0008886)

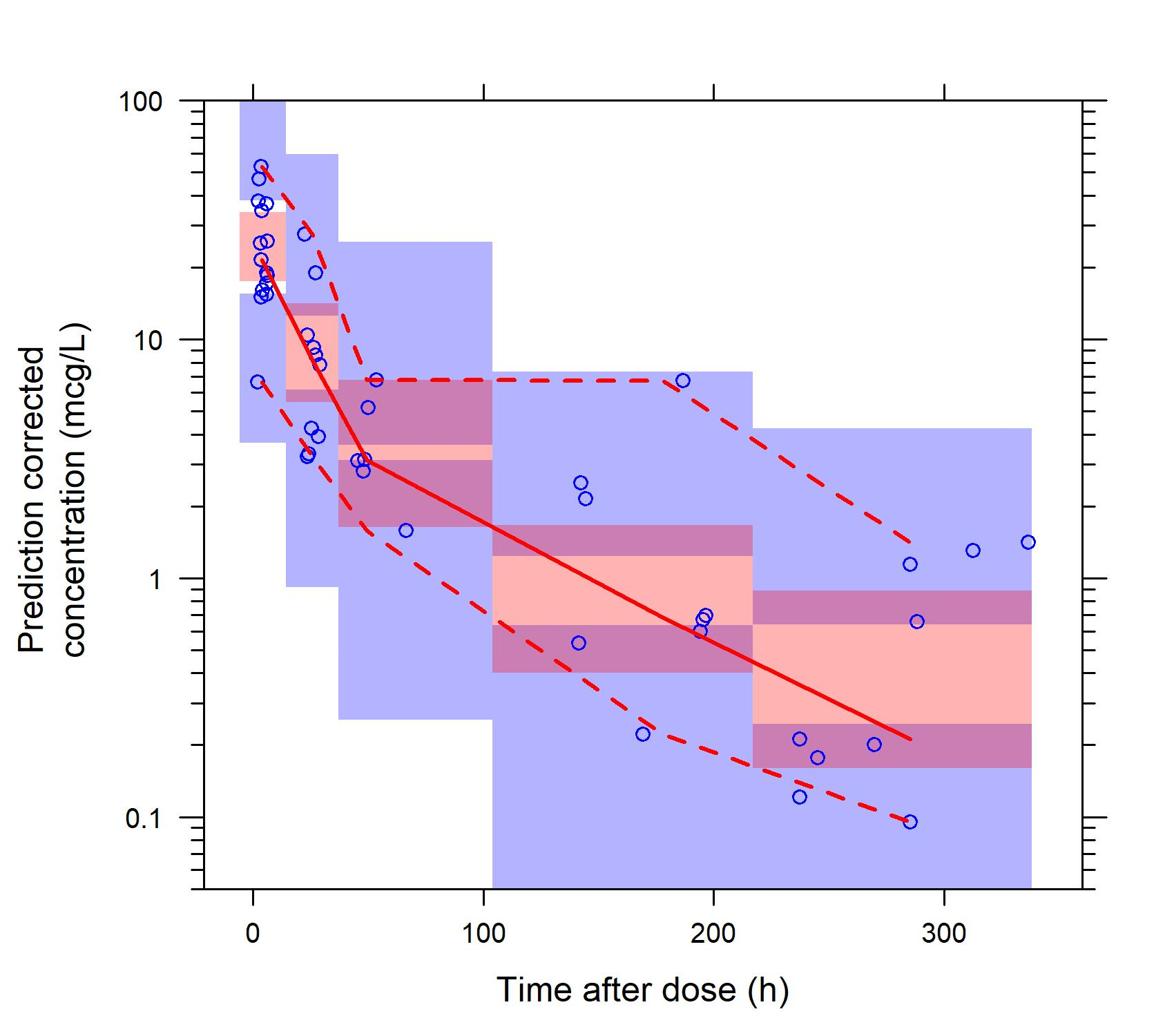

Supplement: S1 Fig — The open circles are observed concentrations. The dashed lines are the 10th and 90th percentiles of the model predictions and the solid line is the 50th percentile. The bands are the 95% CI of the percentiles of the model predictions. (TIF) [file pntd.0008886.s005.tif]

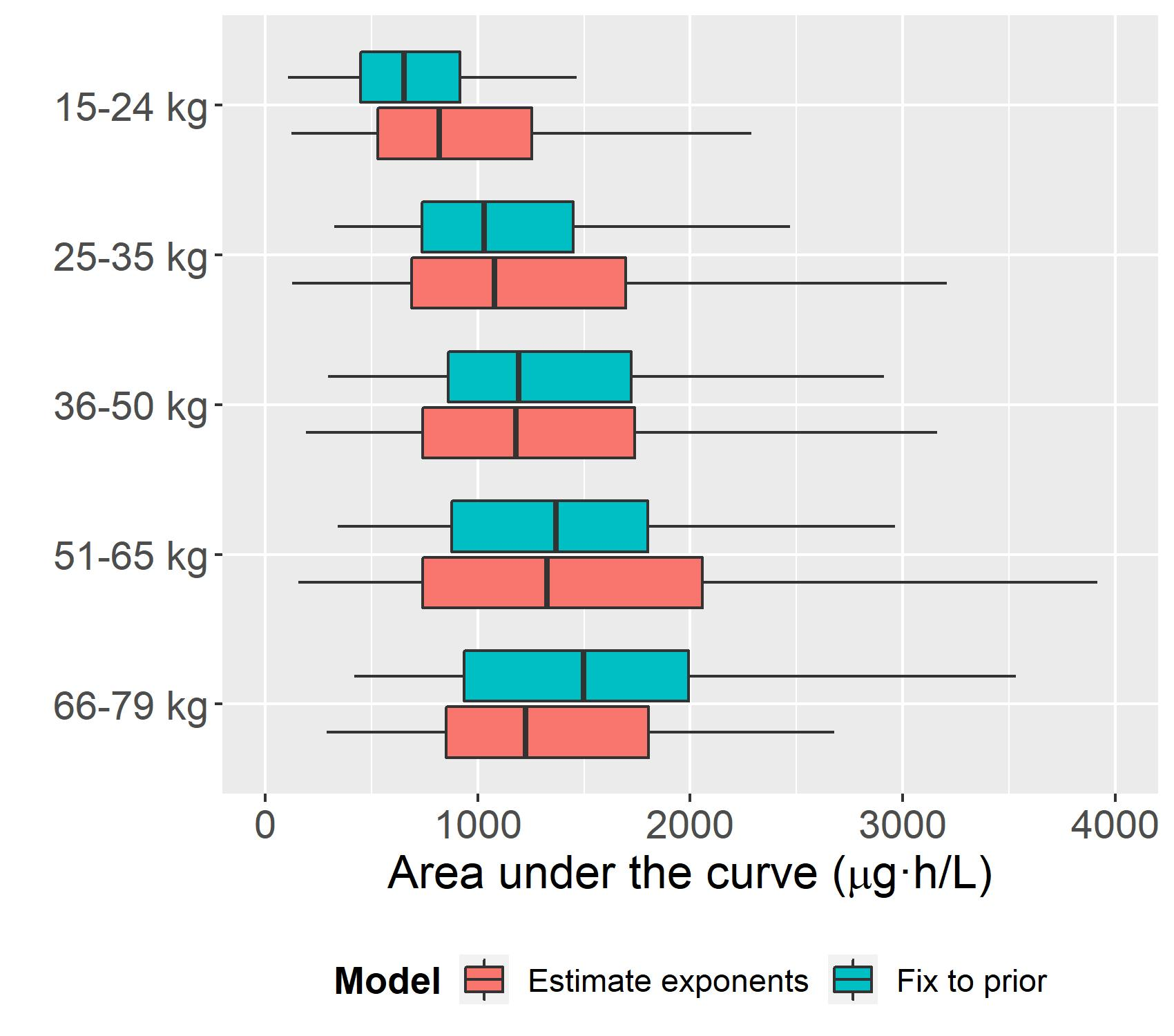

Supplement: S2 Fig — (TIF) [file pntd.0008886.s006.tif]
